# Advances in Musculoskeletal Modeling of the Thoraco-Lumbar Spine: A Comprehensive Systematic Review

**DOI:** 10.1007/s10439-025-03818-8

**Published:** 2025-09-05

**Authors:** Linda Carpenedo, Dominika Ignasiak, Robin Remus, Luigi La Barbera

**Affiliations:** 1 LaBS - Department of Chemistry, Materials and Chemical Engineering “Giulio Natta”, Politecnico di Milano, Piazza Leonardo da Vinci 32, 20133 Milano, Italy; 2 Institute for Biomechanics, ETH Zurich, Zurich, Switzerland; 3 Chair of Product Development, Department of Mechanical Engineering, Ruhr-University Bochum, Bochum, Germany

**Keywords:** Musculoskeletal models, Multibody (MB), Finite element (FE), Coupled models, Hybrid simulation, Spine biomechanics

## Abstract

Understanding spine biomechanics is essential for maintaining posture under static and dynamic conditions, relying on a balance of muscular and gravitational forces. Computational musculoskeletal (MSK) models are increasingly being used in biomechanical research as non-invasive alternatives to in vivo and in vitro methods. Two main MSK modeling strategies are multibody (MB) models, which simplify the spine using rigid vertebrae and intervertebral joints to study muscle recruitment, and finite element (FE) models, which provide detailed tissue representation but often rely on oversimplified loading conditions. Recently, coupled (C) models integrating MB and FE approaches have emerged, though they face technical integration challenges. This literature review examines thoracolumbar MSK modeling methods—MB, FE, and C—to outline current practices, evaluate model capabilities, and inform future research and development. Most reviewed models have been published since 2016, reflecting the growing interest and advances in computational spine biomechanics. While certain modeling choices (e.g., the representation of body weight) are consistent across studies, considerable variability remains in other aspects (e.g., the depiction of muscular architecture, including the selection of muscle groups and the number of fascicles used). Despite being critical for model credibility, validation is often constrained by the limited availability of experimental data. Finally, the review highlights emerging directions such as modeling more complex functional tasks, personalizing anatomical and mechanical properties, and promoting Open Science to enhance reproducibility and collaboration in the field.

**Supplementary Information:**

The online version contains supplementary material available at 10.1007/s10439-025-03818-8.

## Introduction

The biomechanics of the spine, whose primary function is to ensure the maintenance of upright posture under static and dynamic conditions, strongly relies on the balance of muscular forces that counteract weight forces. Understanding these mechanisms is the key to better evaluating the basic functions of the spine in physiological as well as pathological settings. Various structures—bones, soft tissues, and the muscular system—work in harmony to maintain functional equilibrium in healthy conditions [1–5]. Among these, the active contribution of muscles plays a fundamental role in moving the spine while increasing its compressive strength without buckling [1, 6–9]. By doing so, muscles help achieve spinal stability, which is a critical factor for both load-bearing mechanisms and the development of pathological conditions, as it determines the spine’s ability to maintain alignment and resist excessive displacement under physiological and external loads [10]. Several studies reported how the body should always keep inside the ’efficiency cone’ and any deviations from the physiological muscle recruitment and effort; hence, stresses on the spinal structures could trigger the development of spinal disorders, such as lower [11] and upper back pain [12], scoliosis [13] or osteoporotic and traumatic fractures [4, 5]. Notably, low back pain (LBP) is the leading cause of years of healthy life lost due to disability, affecting 619 million people worldwide and posing an economic burden for its treatment [14]. Specifically, the entity of the loads supported by the spine together with their distribution on the different structures play a significant role in the onset and progression of LBP and other spine disorders [11, 15]. Consequently, musculoskeletal (MSK) analysis has become a topic of growing interest, allowing insights into the biomechanical processes that govern healthy and pathological spine configurations. In particular, MSK modeling, i.e., computational modeling with representation of muscle forces, is emerging as a key tool for advancing spine biomechanics. These computational spine models enable the description and prediction of biomechanically relevant variables such as muscle forces, stresses, and strains on bony and ligamentous structures under complex loading conditions, otherwise not achievable with in vivo and in vitro methods. On the one hand, highly invasive in vivo assessments, such as intradiscal pressure (IDP) measurements and electromyography (EMG) [16, 17] are not suitable for routine clinical evaluation. On the other hand, even complex in vitro cadaveric experiments including a few muscles are expensive, time-consuming, and can’t accurately describe the in vivo loading conditions acting on the spine [18].

In this context, computational strategies have become rather established in spine biomechanics, with MSK modeling gaining increasing attention for its ability to overcome experimental limitations and enhance clinical relevance. Multibody (MB) and finite element (FE) models are commonly employed for these evaluations. MB models offer a macroscopic view of the spine, in most cases simplifying vertebrae as rigid bodies and representing intervertebral joints as points with fixed centers of rotation with limited degrees of freedom (DoFs) [19]; however, the possibility to explicitly include muscle fascicles allows the investigation of the recruitment process in very complex tasks, eventually exploiting kinematic data from motion analysis studies. FE models, on the other hand, provide detailed analyses of the loadings on individual spinal tissues, each being described by specific mechanical properties; however, the majority apply simplified boundary conditions (e.g., follower load and/or pure moments) that may not fully capture realistic loadings [20], and only a limited number incorporate the action of muscles. Newer coupled (C) modeling approaches are increasingly being used to overcome the limitations of separate FE and MB models and to combine their capabilities for more holistic considerations in MSK analyses [21, 22].

**Fig. 1:**
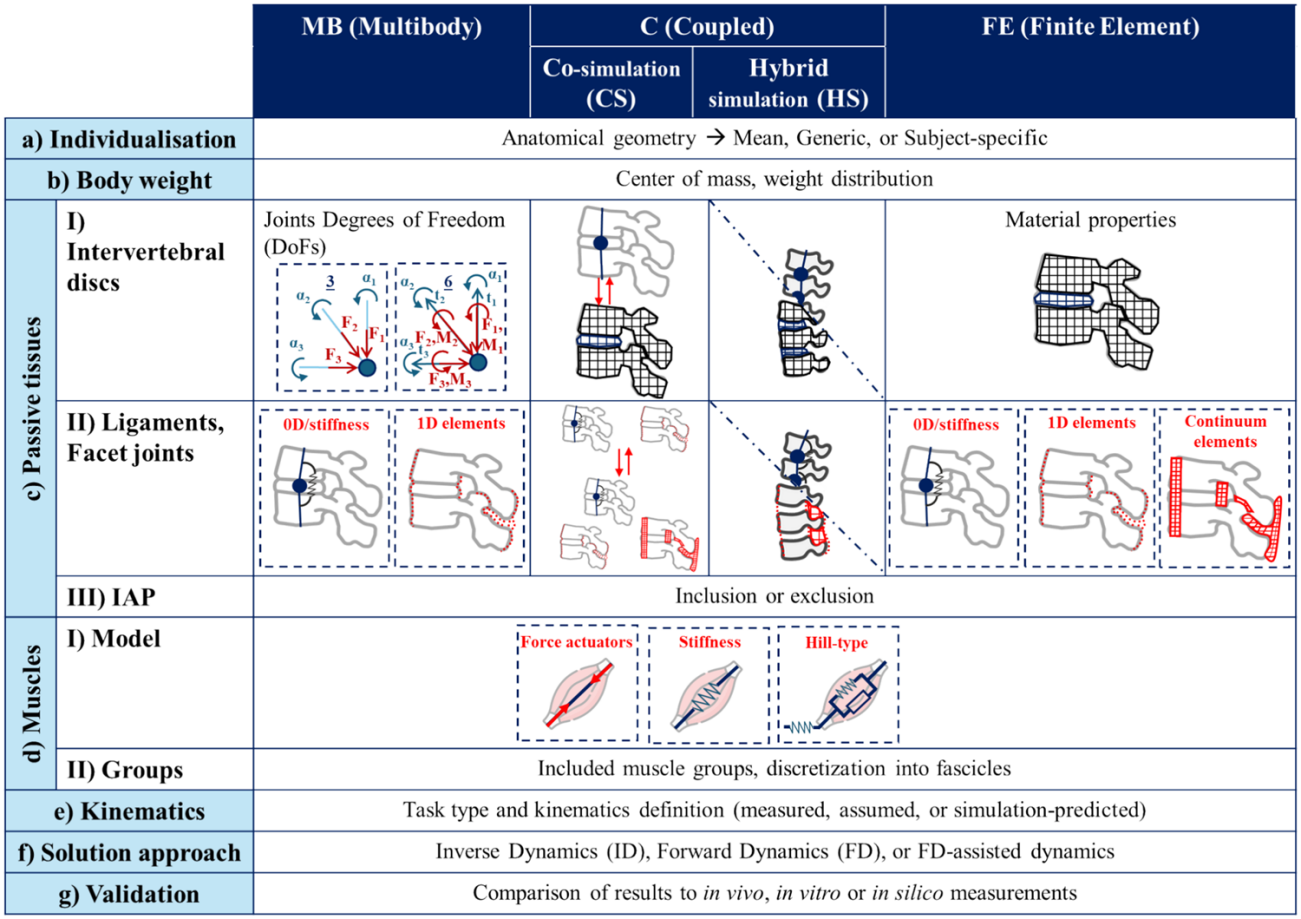
Exemplary workflow for the implementation of a multibody, finite element, or coupled musculoskeletal spine model

The development of accurate modeling frameworks for any computational model relies on several assumptions that should be evaluated by the user to ensure model suitability and credibility for a given ‘context of use.’ The ASME (American Society of Mechanical Engineers) framework provides a structured approach to guide the verification and validation (V&V) processes, ensuring that the models meet the specific needs and applications and thus establish their credibility [23]. Keeping this approach in mind, an ideal workflow for implementing a MSK spine model (Fig. 1) should begin by selecting the modeling approach among MB, FE, and C alternatives. Next, morphological characteristics are defined (Fig. 1a) and body weight (BW) is incorporated (Fig. 1b) by implementing its action with some simplifying schemes. After that, passive structures, i.e., intervertebral disks (IVDs), ligaments, facet joints (FJs), are included (Fig. 1c). Detailed muscle modeling follows (Fig. 1d), accounting for factors like muscle properties and employed groups. Kinematics description follows (Fig. 1e), and the solution approach is chosen (Fig. 1f). Finally, the model undergoes validation through comparisons with data from the literature to ensure its accuracy and reliability (Fig. 1g). Among these steps, some are based on well-established strategies, but several common simplifications still need to be examined to understand their impact on the variables of interest in MSK analysis.

Therefore, this work aims to critically review MSK modeling strategies of the thoracolumbar spine—summarizing MB, FE with muscle representation, and C models—to provide a synthesis of common approaches and a variety of model types and features developed to date. This synthesis aims to inform model selection and development for a given research question in terms of region of interest, level of model complexity and personalization, as well as credibility. It identifies current knowledge gaps and directions for future experimental and computational research.

## Materials and Methods

### Literature Research

PubMed (https://pubmed.ncbi.nlm.nih.gov/) and Scopus (https://www.scopus.com/) databases were retrospectively consulted for the present review between January 2024 and March 2025, with no restrictions on the publication date of the included studies. Preliminary research using the keywords “musculoskeletal AND spinal OR spine AND model AND ((finite AND element) OR multibody OR coupled OR hybrid)” was conducted to obtain the reference database. Only papers written in English, Italian, and German were considered eligible. Specific inclusion and exclusion criteria allowed for the reduction of the number of eligible articles:

- Only articles regarding human spine models were considered;
- Only models that described the action of muscles (and their computing method) were considered;
- Only models including, at least, the thoracolumbar spine were considered;
- Only ‘original’ models proposing for the very first time a new MSK architecture or incorporating major updates to any of the steps presented in Fig. 1 were considered. For instance, simple scaling or morphological adjustments were not considered;
- Translational works where original models were used to study a specific pathology or technique were not included. However, some references were provided as Supplementary Material (Table 1) and discussed to highlight common modeling strategies.

The full texts of the obtained papers were read by one researcher (L.C.), and the resulting tables were double-checked by the coauthors.

### Data Organization and Analysis

All retrieved data were organized based on the modeling approaches: MB, FE, and C models. Among C models, we distinguish two fundamentally different approaches:

1. Co-simulation (CS): a multi-method approach in which MB and FE subsystem models, each operating on separate platforms with their independent solvers, exchange data (i.e., kinematics, muscle forces) during the simulation process. This exchange can be unidirectional, where one simulation runs first and its results are then integrated into the other (e.g., from MB to FE or vice versa), or bidirectional, where data are constantly exchanged [22, 24];
2. Hybrid simulation (HS): an integrated approach that combines FE and MB components within a single simulation model and platform. One solver handles the governing equations for fully dynamically coupled FE-MB simulations.

**Fig. 2:**
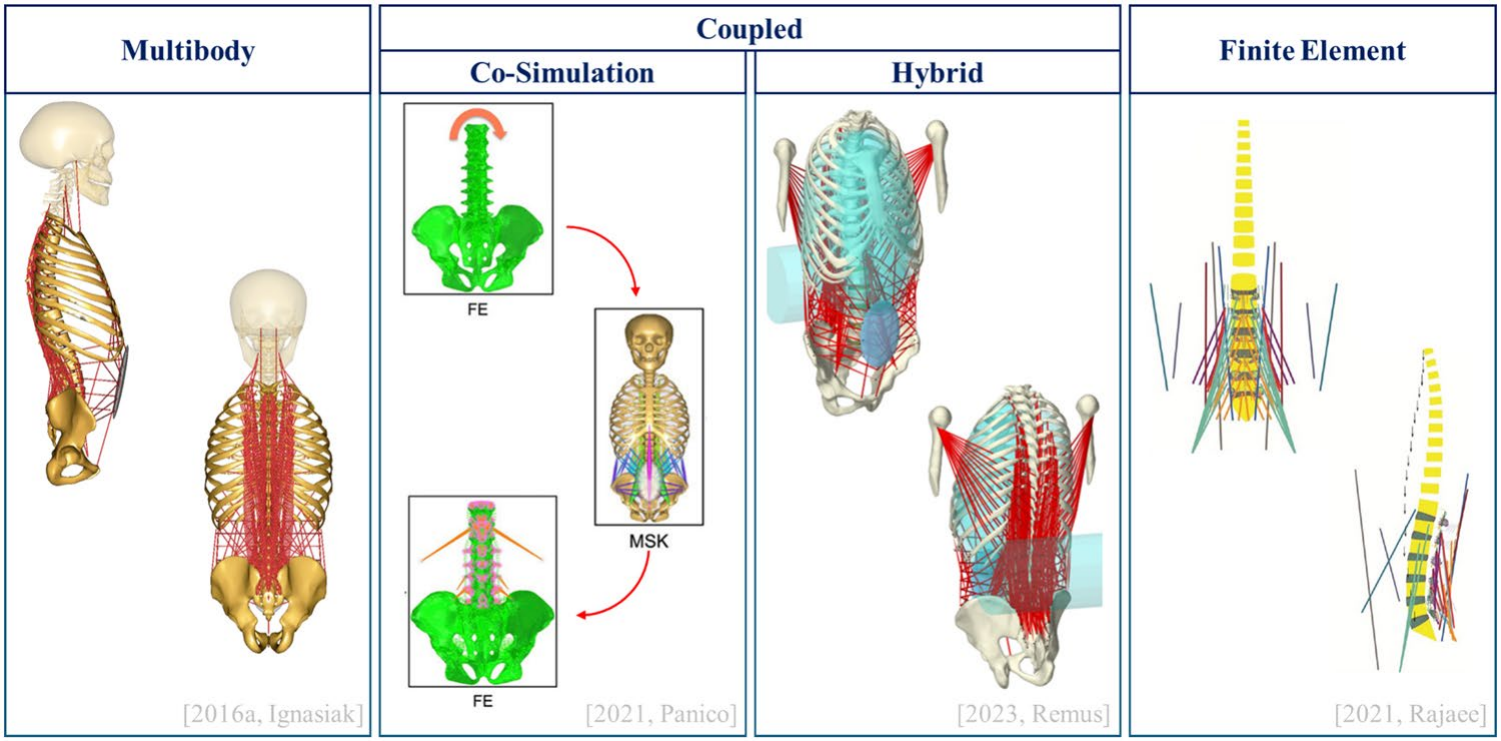
An example for every modeling approach. Multibody image: This figure was published in Journal of Biomechanics, Vol 49, Ignasiak et al., Thoracolumbar spine model with articulated ribcage for the prediction of dynamic spinal loading, Pages 959–966, Copyright Elsevier (2016) [25]. Co-simulation image was taken from Panico et al. [26]. Hybrid model images were provided by Robin Remus. Finite Element image: This figure was published in Journal of Biomechanics, Vol 119, Rajaee et al., novel coupled musculoskeletal finite element model of the spine—critical evaluation of trunk models in some tasks, 110331, Copyright Elsevier (2021) [27].

Representative examples of all these approaches are reported in Fig. 2.

All modeling aspects represented in Fig. 1 were analyzed. General information included:

- Spinal levels: information on the representation of spinal segments or regions;
- Level of individualization: the reference population or the targeted subject that the model represents. Models were classified as ‘*generic’* (i.e., random subject from reference population, chosen to represent the morphological characteristics within a reasonable range), ‘*average’* or ‘*mean’* (i.e., implementing average morphological characteristics, such as height and weight from the normal population, chosen to represent the central properties of the population), or ‘*subject-specific*’ (i.e., representation of a specific subject, chosen to represent her/his individuality) [28] (Fig. 1a);
- Joint modeling, passive tissues, and intra-abdominal pressure (IAP): joint modeling related to the modeling approach is reported: the DoFs of the intervertebral joints for MB models (Fig. 1c-I), the modeling approach for the IVDs in FE models, and both in C models, as well as the modeling of FJs. The description of passive elements used to describe the action of ligaments (Fig. 1c-II) is reported according to classification: through 0D/stiffness (the action of ligaments and/or FJs was either neglected or included in the IVD stiffness matrix), with 1D elements (linear or non-linear spring elements), or with 2D or 3D continuum elements (describing geometry and the material properties of ligaments). Different approaches for modeling IAP are reported as well (Fig. 1c-III);
- BW distributions and center of masses (Fig. 1b);
- Software.

Data on muscle architecture included:

- Dynamics approach. Models were based on three types of dynamics (Fig. 1f): inverse dynamics (ID), forward dynamics (FD), and fD-assisted. ID models used joint kinematics as input, solving an inverse problem to find the combination of forces that could produce that specific motion. Since this approach resulted in multiple possible solutions, optimization criteria—often to minimize specific variables—were used to identify the optimal solution. FD models used kinetic data as input, meaning that muscle forces were directly specified within the model. From these forces, the model calculated the resultant motion. FD-assisted models combined ID and FD approaches, integrating elements of both methods [29]. A tracking-based controller was used to optimize muscle activations to guide a model along a target movement path;
- Muscles’ model: described as simple force actuators, through a stiffness-based relationship or using Hill-type models (Fig.e 1d-I);
- Number of fascicles for each muscle group (Fig. 1d-II).

Finally, the models’ credibility was assessed in terms of validation and reproducibility (Fig. 1f). In accordance with the framework identified by ASME V&V [23] and with the methodology proposed by Stott et al. [30]:

- For each model, validation on kinematic data, muscle forces, and compression forces was reported, when present. Specifically, details on the number and type of comparators used, i.e., in silico, in vivo, or in vitro, the quantity evaluated for the validation, and the corresponding reference for each case were provided. For kinematics, the validation had to be carried out for FD and FD-assisted models only. However, the reference used by ID models as an input was also collected for completeness;
- A model was considered ‘reproducible’ if every key modeling choice (i.e., all the rows from Fig. 1) was properly reported and documented [31], detailed kinematic data were provided for ID models, or FD models included their proper validation. Additionally, both muscular and compressive load validations were required. Reproducibility was assessed using a color-coded system: green indicated complete information, yellow signaled one missing element, and red denoted two or more missing elements. For C models, the clarity of the coupling scheme was also evaluated as an extra criterion. The completeness of muscle architecture data was noted separately (* in Tables 5, 6, 7).

## Results

**Fig. 3:**
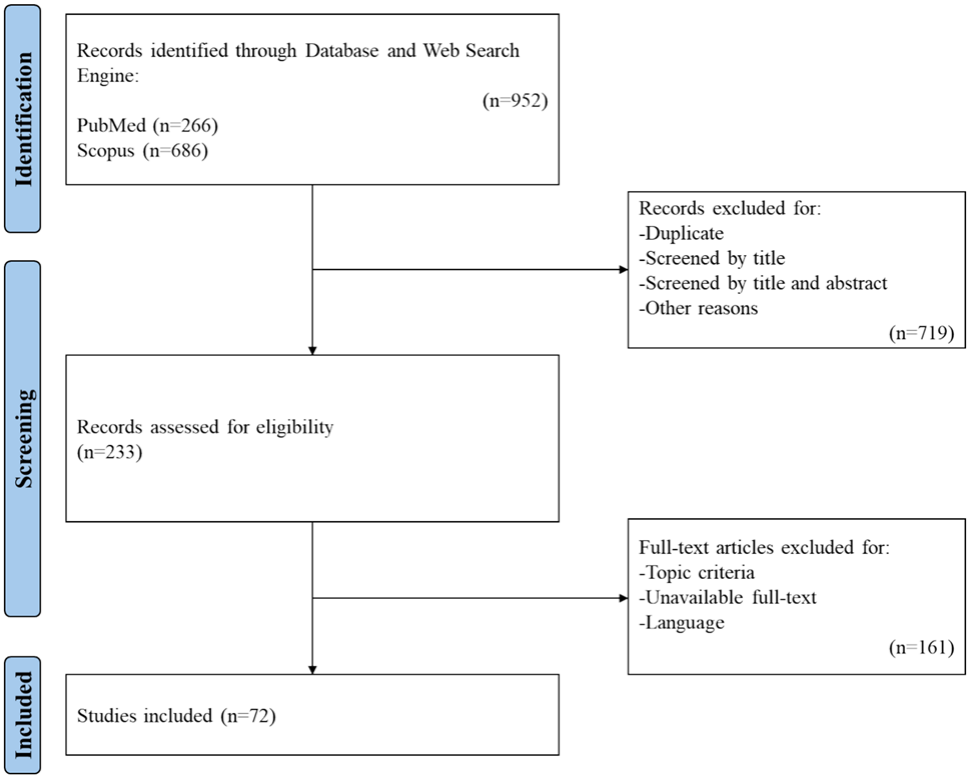
Flow diagram of the literature research procedure

Following a screening process of 952 articles, 44 original models were retained and included in this review. Two further articles were added for validation purposes, and 26 for the discussion to provide an exhaustive overview (Supplementary Material—Table 1). The methodological selection process of useful records according to PRISMA guidelines is graphically outlined in Fig. 3.

Among original models, 26 (59%) were MB [25, 32–56]; 10 (23%) were FE [27, 57–65]; and 8 (18%) were C [26, 66–70]. The first published models dated back to the end of the last century [32, 57, 58], but the majority of models were published after 2016.

### Level of Individualization

Twenty-eight models (61% of all included models) were designed for generic individuals [25–27, 32–45, 47, 48, 51, 54–63, 66–68], 13 (28%) were subject-specific [46, 49, 50, 52, 53, 64, 65, 69–72], and 5 (11%) were mean [34, 35, 37, 38, 61] (Tables 1, 2, 3). The median height of generic models was 1.74 m (range: 1.68–1.8 m) with a median weight of 70 kg (range: 64–78 kg). Although not always reported, the median age of generic subjects was 25 years old (range: 21–78 years), and males were usually chosen (only 4 models represented women [44, 52, 58, 59]). Height and weight matched the average height of 1.75 m (range: 1.67–1.8 m) and weight of 75 kg (range: 64–91 kg) reported for the mean models. Subject-specific models spanned a wider range (height: 1.54–1.8 m; weight: 51–90.3 kg; age: 22–79 years). Subject-specific models were mostly common among MB (25%, 7/28) and were less common among FE models (20%, 2/10) and C models (50%, 4/8). Mean models were used in 14% of MB models (4/28), in 10% (1/10) of FE models, and were absent among C models.

**Table 1:** Details on MB models

MB models									
References	Level of individualization		Joint DoFs	Ligaments	Facet joints	IAP	Ribcage	Software	Notes
	Type (Origin)	H, BW, age, N, sex							
Stokes [32]	Gen (-)	N.a.	3, 6	–	–	–	–	Matlab, SE	–
De Zee [33]	Gen (CT)	1.75 m, 72 kg, –, –, –	3	–	–	–	Lumped w thorax	AnyBody	–
Iyer [34]	Mean (CT)	1.80 m, 91.4 kg, 56.8yrs, 14 M	3	–	–	–	Lumped (load-bearing elements)	SE	–
Han [35]	Mean (CT)	–, –, 50yrs, 55 M + –, –, 49yrs,71F	3	–	–	–	Lumped	SE	–
Christ [36]	Gen (-)	1.70 m, –, –, M	3	–	–	–	Lumped w thorax	OpenSim	–
Han [55]	Gen (scale)	1.80 m, 75 kg, –, M	3	1D tensile-only	–	Upward force	Lumped w thorax	AnyBody	Skull, arms, legs, pelvis
Park [37]	Mean (CT)	1.67 m, 63.67 kg, 35yrs, 2 F + 1 M	3	0D (Joint force)		Upward force	–	Matlab	–
Bruno [39]	Gen (CT)	1.75 m, 78 kg, 25yrs, M	3	–	–	–	Costovertebral pin joints	OpenSim	Lumped head-neck
Huynh [38]	Mean (GEBOD)	1.78 m, 70 kg, –, –, –	3	0D	–	Bushing element	Lumped	LifeMOD	Cervical spine
Khurelbaatar [43]	Gen (CT)	1.70 m, 65 kg, 21yrs, M	6	1D tensile-only	Plane-sphere contacts	–	Costovertebral spherical joints and costosternal cartilages	Recurdyn	Skull, cervical spine
Meng [41]	Gen (-)	1.70 m, 70 kg, –, M	6	0D		Upward force	Lumped w thorax	OpenSim	Upper extremities, lower extremities, lumped head-neck
Rupp [42]	Gen (CT)	1.78 m, 68 kg, –, M	6	1D nonlinear	–	–	Lumped	Demoa	Lumped thoracic–cervic–head–arms part
Senteler [56]	Gen (scale)	1.70 m, 71 kg, –, M	6	0D		–	Lumped w thorax	OpenSim	Head-neck, arms
Dao [44]	Gen (CT)	1.71 m, 64 kg, 78yrs, F	3	–	–	–	Lumped	OpenSim	Head, cervical spine, legs
Ignasiak [25]	Gen (CT)	1.80 m, 75 kg, –, –, –	6	0D		Support + horizontal reaction forces	Costo-vertebral and transverse compound revolute joints; Costosternal articulations 6 DoFs joint	AnyBody	–
Ignasiak [40]	Gen (CT)	2016a, Ignasiak [25]	3	0D		Support + horizontal reaction forces	2016a, Ignasiak [25]; lumped or with updated kinematics	AnyBody	–
Bassani [71]	Sub-s(scale)	1.74 m, 72 kg, 28yrs, M	2007, De Zee [33]						–
Actis [72]	Sub-s (scale)	1.74 m, 70 kg, 45yrs, M; 1.73 m, 73 kg, 25yrs, 8 M; 1.76 m, 74 kg, 43yrs, M	2012, Christophy [36]						Lower body
Malakoutian [45]	Gen (-)	2012, Christophy [36]	6	0D		Upward force	Lumped w thorax	ArtiSynth	–
Bayoglu [46]	Sub-s (ex vivo)	1.54 m, 51 kg, 79yrs, M	3	1D linear torsion springs		–	Costosternal articulations 6 DoFs joints; costo-transverse and -vertebral joints 1DoF revolute joints	AnyBody	Skull, sternum, hyoid, thyrohyoid, clavicles, scapulas, humeri, cervical spine
Higuchi [47]	Gen (CT)	2007, De Zee [33]	3	–	–	–	Costovertebral spherical joint	AnyBody	–
Kamal [48]	Gen (CT)	1.75 m, 68.4 kg, 52yrs, M	3	–	–	–	–	Matlab	–
Guo [51]	Gen (CT)	2015, Bruno [39]	6	1D tensile-only	Plane-sphere contacts	Coupling system w muscles	Lumped	In-house (C+ +)	Arms, head, cervical spine
Fasser [49]	Sub-s(EOS)	–, –, –, 145 (76 F, 69 M)	3	–	–	–	Lumped w thorax	Matlab	-
Favier [50]	Sub-s(MRI)	1.75 m, 67.8 kg, 26yrs, M	3	0D	–	–	Lumped w thorax	OpenSim	Upper and lower extremities
Lerchl [52]	Sub-s (CT)	1.76 m, 65 kg, –, F + 1.73 m, 86 kg, -, M	3	1D nonlinear	–	–	Lumped w thorax	Simpack	Lumped head-neck w thorax
Meszaros-Beller [53]	Gen + Sub-s (CT)	Generic: 1.78 m, 81.5 kg, –, M	6	1D	Capsular ligament	–	Lumped w thorax	Demoa	-
Mo [54]	Gen (-)	1.80 m, 73.9 kg, –, –, –	6	1D	1D (torque)	–	Lumped w thorax	OpenSim	Lumped thorax, cervical, ribcage, scapulae, head

**Table 2:** Details on FE models. Gen = generic, Sub-spec = subject-specific

FE models								
References	Level of individualization		IVD definition	Ligaments	Facet joints	IAP	Ribcage	Software
	Type	H, BW, age, N, sex						
Kiefer [57]	Gen (CT)	N.a.	Euler beam elements	–	–	–	–	Abaqus
Kiefer [58]	Gen (CT)	1.70 m, 68 kg, –, F	Beam elements	–	–	–	–	Abaqus
Arjmand [59]	Gen (CT)	1998, Kiefer [58]	Beam elements	0D		–	–	Abaqus
Kim [60]	Gen (-)	1995, Stokes [32]	Beam elements	0D		–	–	N.a.
Kim [61]	Mean (CT)	–, –, 50yrs, 55 M + –, –, 49yrs, 71 F	Solid elements with nonlinear elastic property	0D (Longitudinal Ligaments) + 1D (tensile-only)	1D (nonlinear compression-tension)	–	Lumped w thorax	LS-DYNA
Ghezelbash [62]	Gen (CT)	1.74 m, 68 kg, –, –, –	Quadratic shear deformable beams w nonlinear properties	0D		–	–	Abaqus
Toumanidou [63]	Gen (CT)	–, 70.8 kg, –, –, –	Uniaxial hypoelastic anulus fibers; Neo-Hookean incompressible anulus matrix; Mooney–Rivlin incompressible nucleus	1D (Hypoelastic formulation)	Tension: isotropic linear elastic + Compression: hypoelastic	–	–	Abaqus
Ghezelbash [64]	Sub-s (scale)	1.8 m, 73 kg, 30yrs, M + 1.78 m, 73 kg, 25yrs, F + 1.74 m, 72 kg, 45yrs, M + 1.75 m, 68 kg, 52yrs, M	Beam elements	0D		Force at T11	–	Abaqus
El Bojairami [65]	Sub-s (MRI)	1.728 m, 65 kg, 22yrs, M	Volumetric deformable anulus, shell structure enclosed with hydrostatic elements nucleus	–	–	–	-	ANSYS
Rajaee [27]	Gen (CT)	1.74 m, 68 kg, –, –, –	Anulus layers, disk nucleus as isotropic bulk (w fibers, w Mooney-Rivlin material) + fluid-filled cavities	1D (tensile-only)	Contact facet surfaces	–	–	Abaqus

**Table 3:** Details on C models

C models											
References	Level of individualization		Joints DoFs/IVDs definitions	Ligaments	Facet joints	IAP	Rib cage	C type	Direction	Software	Notes
	Type	H, BW, age, N, sex									
Liu [66]	Gen (CT)	1.68 m, 70 kg, –, M	3	1D tensile-only	–	Cylinder w constant volume and pressure	–	CS	Uni: MB→FE muscle forces (kinematics iteratively adjusted)	AnyBody	Lumped head-neck, upper arms, thorax
			Hyperelastic Mooney-Rivlin + collagen fibers 1D nonlinear	1D tensile-only	Shell elements + frictionless surface-to-surface contact	Boundary conditions derived MB model				Abaqus	–
Khoddam-Khorasani [68]	Gen (CT)	–, –, 65yrs, M	Shear-deformable beams	–	–	–	–	CS	Uni: MB→FE muscle forces (joint properties iteratively adjusted)	Abaqus	–
			Isotropic anulus bulk (w collagen fibers) + fluid cavities + 1D nonlinear lamellae	1D tensile-only	Frictionless surface-to-surface contacts	–				Abaqus	–
Liu [67]	Gen (-)	1.68 m, 70 kg, –, M	3	1D tensile-only	Facet contacts	Cylinder w calculated (simulation) pressure	Lumped w thorax	CS	Uni: MB→FE (kinematics iteratively adjusted)	AnyBody	Lumped head-neck,upper arms
			Hyperelastic Mooney-Rivlin	1D nonlinear	Frictionless surface-to surface contact	Boundary conditions from MB model	–			Abaqus	–
Favier [73]	Sub-s (MRI)	1.75 m,67.8kg, 26yrs, M	2021a, Favier [50]					CS	Uni: MB→FE joint and muscle forces	OpenSim	–
			Linear elastic material	–	–	–	–			Abaqus	–
Kumaran [74]	Sub-s (CT)	–, –, 55yrs, –	2015, Bruno [39]					CS	Uni: FE for RoMs→MB muscle forces→FE	OpenSim	Lumped head-neck
			Anulus composite solid, linear elastic nucleus	Truss elements (tensile-only)	3D gap elements, non-linear soft contact	–	–			Abaqus	–
Panico [26]	Gen (-)	2016b, Ignasiak[40]	2016b, Ignasiak [40]	2016b, Ignasiak [40]	2016b, Ignasiak [40]	2016b, Ignasiak [40]	Costo-vertebral and transverse compound revolute joints	CS	Uni: FE for RoMs →MB muscle forces →FE	AnyBody	–
			Anulus w 1D collagen fibers (nonlinear)	1D spring	1D nonlinear	–	–			Abaqus	–
Remus [69]	Sub-s (CT)	1.80 m, 90.3 kg, 38yrs, M	Hyperelastic Mooney-Rivlin anulus (w 1D collagen fibers nonlinear), quasi-incompressible Yeoh nucleus	1D tensile-only	Frictionless, nonlinear elastic cartilage	Force applied to the thorax, dynamically calculated from abdominal muscles	Lumped w thorax	HS	–	ArtiSynth	–
Xu [70]	Sub-s (CT, MRI)	1.69 m, 67.85 kg, –, –, –	6	1D tensile-only	1D (torque)	–	Lumped w thorax	CS	Uni: MB→FE muscle forces	OpenSim	–
			Hyperelastic Mooney-Rivlin + nonlinear elastic fibers	3D	Nonlinear elastic joint capsules	–	–			LS-DYNA	–

*Gen* = generic, *Sub-spec*  subject-specific, *Uni* = unidirectional coupling, * RoM* = range of motion

### Spinal Levels

**Fig. 4:**
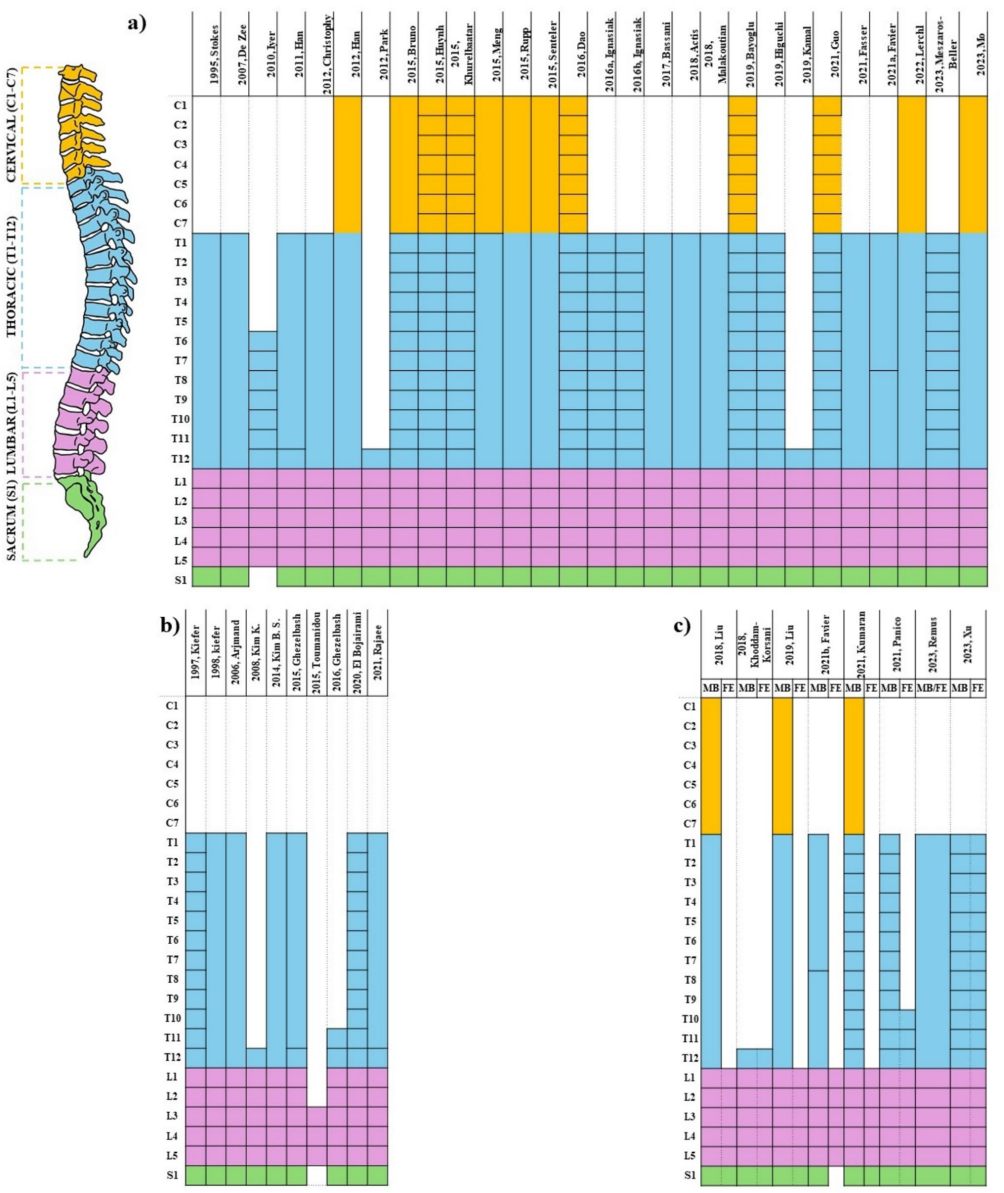
Levels included in **a** MB models, **b** FE models, **c** C models. Separated rectangles represent individual bodies, while if some regions are represented as lumped, the corresponding rectangles are merged together

The majority of the reviewed models (31, 67%) represented the lumbar spine only, with the thoracic region either represented as a lumped rigid body [27, 32, 33, 35, 36, 41, 42, 45, 49, 50, 52, 54–56, 58, 59, 61, 62, 66, 67, 69, 71, 72] or omitted [34, 37, 48, 60, 63, 64, 68, 73]. The detailed thoracic levels were provided in 14 (30%) models [25, 26, 38–40, 43, 44, 46, 47, 51, 53, 57, 65, 70]. The cervical region was included in 14 (30%) models, either as a single lumped segment (9, 20%) [39, 41, 42, 52, 54–56, 66, 67] or as an individual vertebrae (5, 11%) [38, 43, 44, 46, 51] (Fig. 4a, b, c). The ribcage was included in 61% of the original models, either as a lumped segment (21 models) [33–36, 38, 41, 42, 44, 45, 49–56, 61, 67, 69, 70] or as an articulated system of ribs and sternum (7 models) [25, 26, 39, 40, 43, 46, 47] (Tables 1, 2, 3).

### Joint Modeling, Passive Soft Tissues, and IAP

#### MB Models: Joints DoFs

Fifteen (15/26, 58%) original MB models used 3 DoFs intervertebral joints [33–39, 44, 46–50, 52, 55] and 10 (38%) used 6 DoFs joints [25, 40–43, 45, 51, 53, 54, 56]. 1 (1%) used both to evaluate the different effects of these modeling assumptions [32]. Ligaments were neglected in 10 (38%) models [32–36, 39, 44, 47–49], included as part of the IVD stiffness properties in 8 models (31%) [25, 37, 38, 40, 41, 45, 50, 56], and represented as 1D elements in 8 models (31%) [42, 43, 46, 51–55]. As for FJs, 15 (58%) models neglected their action [32–36, 38, 39, 42, 44, 47–50, 52, 55, 56] and 6 (23%) included it in the IVD properties [25, 37, 40, 41, 45]. Only 2 (8%) of the MB original articles accounted for their contact interactions [43, 51]. As for IAP representation, 8 (31%) models included its action, and the majority of them (7) simplified it through an upward force [37, 38, 41, 45, 55] sometimes with a simultaneous horizontal one [25, 40], while 1 proposed an iterative computational scheme that dynamically adapted IAP action due to changes in muscle activation, geometry, and movement [51] (Table 1).

#### FE Models: Continuous Vs. Discrete Elements

IVDs were mostly represented as beam elements with 3D stiffnesses in 7 (7/10, 70%) models [57–62, 64], while two of the most recent articles modeled IVDs to represent fibers in the anulus and the incompressibility of the nucleus [27, 63], and 1 used volumetric bodies [65]. Ligaments were included in the IVD stiffness properties in 4 (40%) models [59, 60, 62, 64], represented as 1D elements in 3 (30%) [27, 61, 63], and neglected in 3 (30%) [57, 58]. As for FJs, the 2 most recent articles presented a detailed description of their interaction type [27, 63]. Two models included the action of IAP [64, 65] (Table 2). No FE MSK models described the costo-vertebral joint.

#### C Models

Seven (7/8, 88%) C models were of CS type, and a unidirectional data exchange was performed in all, with an iterative scheme being exploited by 3 models [26, 66, 67]. In two of the CS models, the precise information that was used for coupling was not explicitly described [68, 70]. Finally, only 1 model was of HS type [69]. In C models, the MB and FE components typically adopted modeling strategies similar to those previously described for standalone MB and FE models. In particular, 3 [66, 67] or 6 [26, 70] DoFs joints or shear deformable beams [68] were used in the MB component. In the FE part, a trend toward more complex IVD modeling strategies was identified. Ligaments were represented as 1D elements in both the MB and FE counterparts in 4 (50%) models [26, 66, 67, 69], while their action was represented through 3D elements in the FE counterpart in 1 model [70]. As for FJs, in the FE part of C models, frictionless surface-to-surface contacts were used to represent the interaction of FJs in 5 (63%) original models [66–69, 74]. IAP was represented as an upward force [26], through a pressurized cylinder [66] or dynamically calculated [67, 69] (Table 3).

### Body Weight

To represent complex in vivo loading conditions, MSK models need to account for the effects of BW at each spinal level. Thirty-seven models (80%) represented BW by applying an eccentrically concentrated force that represented the contribution of a certain vertebral level, together with the surrounding tissues [25–27, 33, 34, 36, 37, 39–41, 43–59, 62–64, 68–74]. Pearsall et al. [75] was the primary reference and was cited by 22 works [26, 27, 36, 37, 39–41, 43, 45, 46, 48, 49, 53, 54, 56, 59, 62–64, 68, 70, 74]. The others used a similar approach but with different references from the literature [25, 33, 34, 44, 47, 57, 58, 69, 71, 72]. Only 3 original MB models provided a personalized alternative to this scheme [49, 50, 52]. Two MB [35, 42] and 2 FE [61] (9%) models applied a concentrated BW in a calculated center of gravity. One MB [32] model neglected the action of BW, while 1 MB [38], 1 FE [65], and 2 C [66, 67] models didn’t provide information on the employed scheme.

### Active Structures: Muscles

**Fig. 5:**
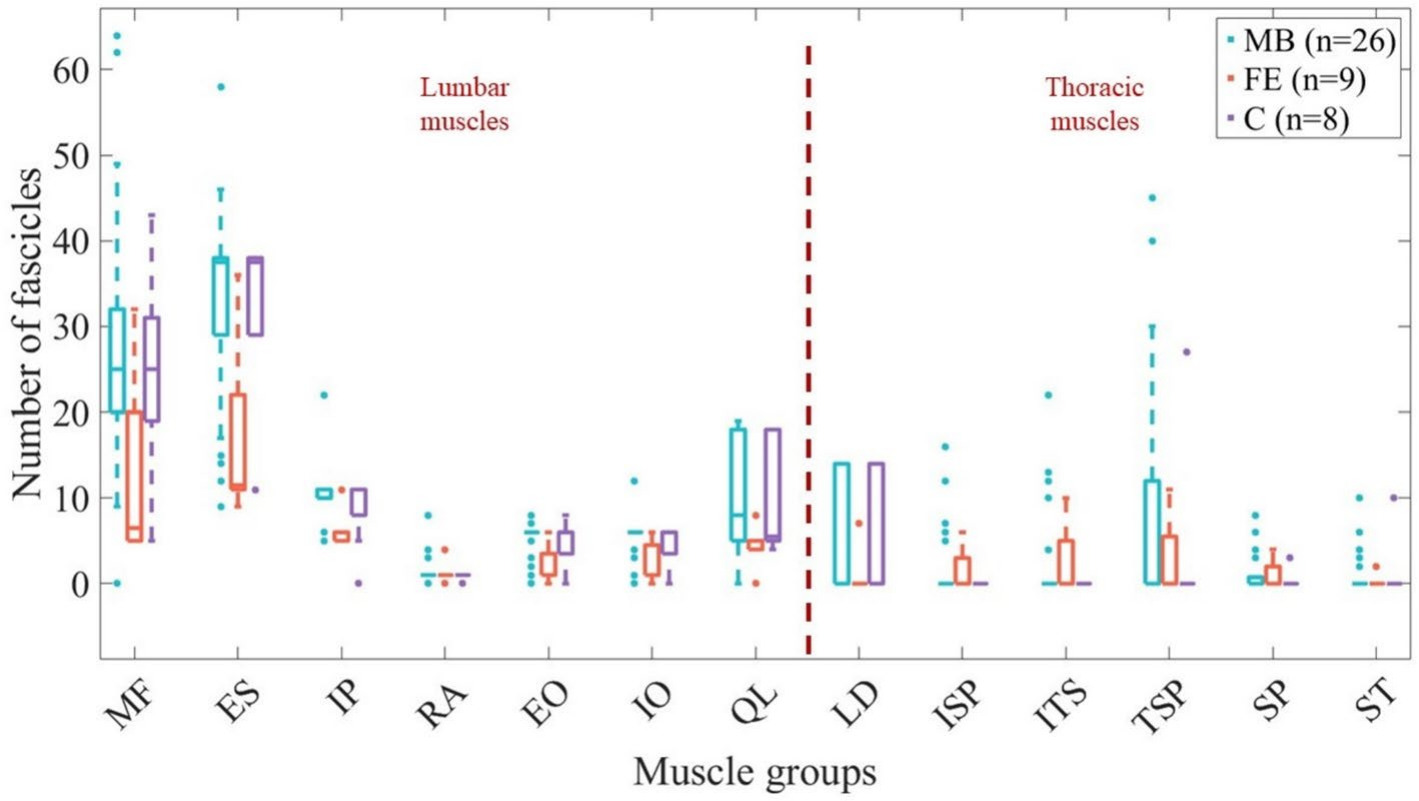
Box plots for the number of fascicles implemented for each muscle group by the different modeling approaches. *MF* multifidus, *ES* erector spinae, *IP* iliopsoas, *RA* rectus abdominis, *EO* external oblique, *IO* internal oblique, *QL* quadratus lumborum, *LD* latissimus dorsi, *ISP* interspinales, *ITS* intertransversarii, *TSP* transversospinalis, *SP* spinalis, *ST* serratus

Muscles were modeled as force actuators in 22 (50%) original articles [25, 26, 32–35, 37, 38, 40, 43, 46–48, 52, 55, 57, 60, 61, 66–68] (15 MB, 3 FE, and 4 C), through stiffness in 5 (11%) [27, 58, 59, 62, 64] FE models and through a Hill-type model in 17 (39%) [36, 39, 41, 42, 44, 45, 49–51, 53, 54, 56, 69, 70] (13 MB and 4 C). One original FE model proposed a constitutive model for muscles’ behavior (1D nonlinear) [63] and 1 FE model represented muscles as fluid-filled pressurized tissues acting with an intramuscular pressure [65] (Table 4). MB models developed for MSK modeling included more fascicles than FE models; moreover, spine models extending also to the thoracic segment implemented a higher number of fascicles [25, 26, 39, 40, 46, 47, 53] (Fig. 5, Supplementary Material—Tables 2, 3, 4).

**Table 4:** muscle information for MB, FE, and C models

References	Approach	Optimization scheme	Muscle model	Fascicles (both sides)
**MB models**				
Stokes [32]	SE	Max moment	Force actuators	132
De Zee [33]	ID	Min/max	Force actuators	154
Iyer [34]	SE	Min/max, min sum of cubed muscle stresses	Force actuators	158
Han [35]	SE	Min joint loads	Force actuators	232
Christophy [36]	–	–	Hill-type	238
Han [55]	ID	Min muscle activations	Force actuators	516
Park [37]	ID	Min muscle stress and shear forces	Force actuators	180
Bruno [39]	ID	Min sum of cubed muscle stresses	Hill-type	248
Huynh [38]	ID	–	Force actuators	154
Khurelbaatar [43]	ID	Min sum of cubed muscle stresses	Force actuators	218
Meng [41]	ID	Min sum of muscle and actuator activations squared	Hill-type	238
Rupp [42]	FD	–	Hill-type	124
Senteler [56]	ID	Min sum of squared muscle activations	Hill-type	238
Dao [44]	ID	Min muscle activations (muscle excitation)	Hill-type	152
Ignasiak [25]	ID	Min sum of cubed muscle stresses	Force actuators	454
Ignasiak [40]	ID	Min sum of cubed muscle stresses	Force actuators	508
Bassani [71]	ID	Min/max	Force actuators	154
Actis [72]	ID	Min sum of squared muscle activities	Hill-type	238
Malakoutian [45]	FD-assisted	Min sum of weighted square of muscle activations + tracking	Hill-type	210
Bayoglu [46]	ID	Min sum of cubed muscle stresses	Force actuators	552
Higuchi [47]	ID	Min sum of cubed muscle stresses	Force actuators	328
Kamal [48]	ID	Min sum of cubed muscle stresses	Force actuators	92
Guo [51]	FD	Solution considered physiological cross-sectional areas	Hill-type	214
Fasser [49]	ID	Min sum of squared muscle activities	Hill-type	230
Favier [50]	ID	Min sum of squared muscle activities	Hill-type	238
Lerchl [52]	ID	Min sum of cubed muscle activities	Force actuators	206
Meszaros-Beller [53]	FD	Equilibrium point based on muscle fiber lengths	Hill-type	294
Mo [54]	FD	–	Hill-type	238
**FE models**				
Kiefer [57]	ID	Min muscle forces	Force actuators	4
Kiefer [58]	ID	Min compressive loads	Stiffness K = qF/l	60
Arjmand [59]	ID	Min sum of cubed muscle stresses	Stiffness K = qF/l	56
Kim [60]	ID	Min sum of squared internal joint loads (forces and moments)	Force actuators	234
Kim [61]	ID	Min internal joint loads joint forces and moments)	Force actuators	232
Ghezelbash [62]	ID	Min sum of cubed muscle stresses	Stiffness K = qF/l	56
Toumanidou [63]	FD	–	1D nonlinear	46
Ghezelbash [64]	ID	Min sum of squared muscle stresses	Stiffness K = qF/l	126
El Bojairami [65]	[76]: Min displacement	[65]: –; [76]: min muscle effort, min IVD compression, min spine displacement, min or max intramuscular pressure	Intra-muscular pressure	Not applicable
Rajaee [27]	ID	Min sum of cubed muscle stresses	Stiffness K = qF/l	56
**C models**				
Liu [66]	ID	Min sum of square of the ratios muscle force to muscle strength	Force actuators	188
Khod.-Khorasani [68]	ID	Min sum of cubed muscle stresses	Force actuators	56
Liu [67]	ID	Min sum of square of the ratios muscle force to muscle strength	Force actuators	154
Favier [73]	ID	Min sum of squared muscle activities	Hill-type	238
Kumaran [74]	ID	Min sum of cubed muscle stresses	Hill-type	248
Panico [26]	ID	Min sum of cubed muscle stresses	Force actuators	508
Remus [69]	FD-assisted	Min sum of weighted squares of muscle activations + tracking	Hill-type	258
Xu [70]	FD	–	Hill-type	162

*SE* = static equilibrium, *ID* = inverse dynamics, *FD* = forward dynamics

Lumbar models had consistent muscle groups and fascicle architecture: multifidus, erector spinae, iliopsoas, rectus abdominis, external oblique, internal oblique, and quadratus lumborum were always present. The number of fascicles could span from very realistic representations [36, 45, 50, 61, 77] to simplified ones merging fascicles attached at the same levels [43, 48, 58, 68]. For instance, the number of fascicles for quadratus lumborum varied from 3 [43] to 19 [52]. Thoracic models had inconsistent muscle groups, with C and FE models rarely extending to this region [26, 60, 61, 66, 67]. The tranversospinalis group was the most commonly included, appearing in 6 out of 12 models with thoracic levels [25, 40, 43, 46, 47, 53], but the number of fascicles varied among these models. Spinalis was also frequently represented (in lumbar models as well); however, the number of fascicles could vary from 1 [58] to 8 [53]. The remaining muscles, in particular interspinales, intertransversarii, and serratus groups were never described by C models, while increasing in MB models compared to FE ones (Fig. 4). In the only FE model representing muscles as 3D tissues, multifidus, psoas major, longissimus, latissimus dorsi, and intertransversarius were included [65].

### Kinematics Strategy

Thirty-four (74%) models were ID-based [25–27, 33, 37–41, 43, 44, 46–50, 52, 57–62, 64–68, 71, 72], and minimized muscle stresses to find the optimal muscle solutions. Six (13%) models were based on FD [42, 51, 53, 54, 63, 70], while 2 (4%) used FD-assisted algorithms [45, 69]. Finally, 3 (7%) models among the MB ones were based on static equilibrium approaches [32, 34, 35] (Table 4).

In C models with CS approach, data are exchanged between the subsystem models via a coupling interface. All the CS models were unidirectional coupled and muscle data from the upstream MB model was transferred to the downstream FE model [26, 66–68, 70]. In 3 of these 7 cases, iterative adjustments to find coherence among the MB and FE approaches were applied [66–68]. In 1 C model, a HS approach was used with the simulation platform ArtiSynth [69].

### Modeled Tasks

Tasks simulated with the models were usually chosen based on the available reference data in the literature. Thirty-six out of 46 models (78%) were used to analyze standing [25, 27, 33–35, 37, 39, 42, 45–57, 59–64, 66–71, 73] and different amplitudes of flexion [25–27, 34, 36, 39–44, 46, 47, 49–56, 59, 61, 62, 64–69, 71–74].

Six original articles characterized spinal stability by simulating perturbations [57, 58] or studying the stability margins of the models [48, 59, 60, 69].

### Credibility Assessment: Validation and Reproducibility

Information on the prescribed kinematics was incomplete in 11 ID-based models [33, 35, 37, 38, 43, 47, 48, 55, 57, 58, 61]. In FD or FD-assisted-based models, kinematics was validated in comparison with in vivo, in vitro, or in silico data. As for muscle forces, most studies qualitatively compared predicted muscle forces to in vivo EMG data from superficial electrodes [34, 39, 52], sometimes in terms of the percentage of maximum voluntary contraction [54, 59, 64, 72]. Compressive forces predicted by the models were compared to in vivo IDP measurements [27, 33, 34, 37–39, 41–43, 45–47, 50, 52, 54, 59, 60, 64–66, 68, 69, 71– 72]. IDP comparison was often based on an indirect estimation (adjustments with the disk area only or together with a correction factor). Only 6 articles could propose a direct comparison of their results to the experimental ones [27, 44, 65, 67, 69, 70].

As for reproducibility, in most cases, the condition was not satisfied because of the unavailability of the input data for kinematics in ID-based models. The complete dataset of muscle architecture was presented by 6 models only [32, 36, 46, 48, 58, 69], while 3 AnyBody-based models can be consulted by requesting a free trial license [25, 33, 40] (Tables 5, 6, 7).

**Table 5:**
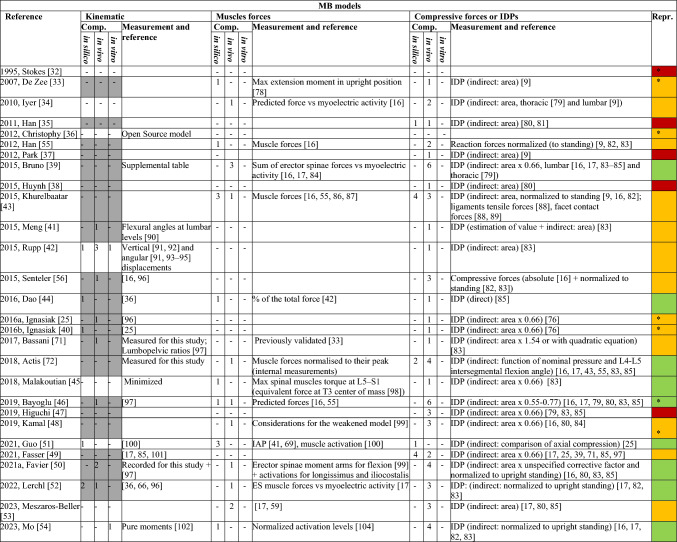
Information on the validation process for MB models

The number of in silico, in vivo, and in vitro comparators is indicated. Grey cells indicate that data were used as input in the model, and not checked for validation. Green cells indicate complete information, yellow ones signal one missing element, and the red cells denote two or more missing elements. The * in the reproducible column indicates that the complete dataset on muscle coordinates is provided in the work

**Table 6:**
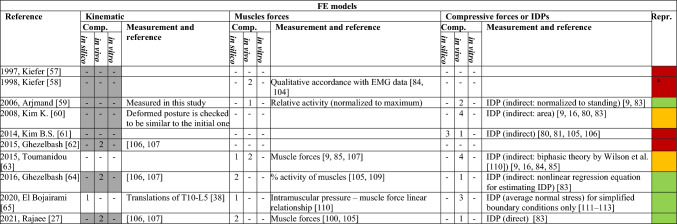
Information on the validation process for FE models

The number of in silico, in vivo, and in vitro comparators is indicated. Grey cells indicate that data were used as input in the model, and not checked for validation. Green cells indicate complete information, yellow ones signal one missing element, and the red cells denote two or more missing elements. The * in the reproducible column indicates that the complete dataset on muscle coordinates is provided in the work

**Table 7:**
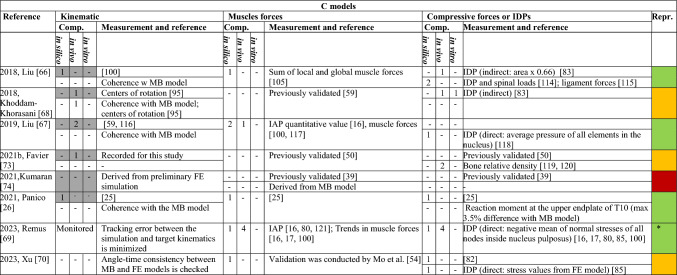
Information on the validation process for C models

The number of in silico, in vivo, and in vitro comparators is indicated. Grey cells indicate that data were used as input in the model, and not checked for validation. Green cells indicate complete information, yellow ones signal one missing element, and the red cells denote two or more missing elements. The * in the reproducible column indicates that the complete dataset on muscle coordinates is provided in the work

## Discussion

This review critically examined existing MSK modeling strategies of the thoracolumbar spine, focusing on MB, FE, and C models with muscle representation. Forty-four original models were found, with different levels of detail. By synthesizing the diversity of model types, complexities, and applications, this work aims to guide model selection and development while highlighting current limitations and future research directions.

### Overview of Current MSK Models of the Spine: Aligning the Strategy to Study Objectives

The systematic review demonstrated that a multitude of modeling approaches are available for studying spine biomechanics by integrating the action of muscles. From holistic muscle force evaluations to detailed tissue-level analyses, these models provide researchers with tools to investigate specific aspects of spine function and its biomechanics. The choice of model depends on the study objectives, balancing detail, computational demand, and biomechanical insights.

#### MB Models at the Organ Level, FE at the Tissue Level, C Integrate Both

The present review categorized results based on modeling approaches. MB models were the most common in MSK research and benefited from open-source availability [36, 39, 41, 44, 45, 50, 54, 69, 122]. These models provide a holistic perspective on spine biomechanics and are suitable for evaluating overall biomechanical aspects at the macroscopic body level (i.e., joint and muscle estimates) [22]. FE models, on the other hand, allow the detailed description of the morphology and heterogeneous mechanical properties of underlying spinal structures at the tissue level, estimating their contribution in terms of load-sharing and providing details on stress-strain distributions across spinal structures [66]. However, FE models are computationally demanding and require simplifying assumptions for muscle force implementation [27, 61, 66]. C models emerged in response to the limitations of traditional MB and FE models, aiming to combine their respective strengths: accurate muscle force estimation for MB and detailed structural analysis for FE [22]. As this represents a relatively new framework, we introduced a clear terminology to distinguish CS and HS methods. Other approaches are also possible, such as merging subsystem equations into a single model for monolithic solving (distributed modeling) or dividing a model into subsystems within one tool and solving them with specialized solvers (distributed simulation) [24]. However, no implementation of these techniques was found among MSK thoraco-lumbar spine models.

Despite their promise, the reliability of C models depends on the effective integration of their components. Their application is also highly sensitive to initial conditions and study objectives, often requiring specific simplifications and technical implementations. As a result, solutions tend to be tailored to individual problems, involving complex computational processes, data exchanges, and boundary conditions.

#### Spinal Levels and Rib Cage

The spinal levels should be selected based on study objectives. For instance, a model targeting LBP should focus on the L1-S1 segment to precisely assess the kinematics and loads in the lumbar spine that could lead to the pathology or arise from it [123]. Thoracolumbar studies require thoracic and lumbar levels, while broader investigations benefit from extended models, but only a few options of this kind were available in the literature [38, 43, 46]. Ribcage representation also depends on study goals. Ignasiak et al. proved that a rigid thorax is sufficient for caudal lumbar load estimation, but a fully articulated ribcage is needed for accurate kinematics and load assessment at upper lumbar levels [40]. In vitro tests confirmed the role of the ribcage in stabilizing the thoracic spine [124, 125], thus making its detailed representation essential for studying the thoracic region and thoracolumbar junction.

### Choosing the Level of Model Complexity

MSK models were found to vary widely in complexity, reflecting the diverse methods and features developed. While advancements have expanded the range of available tools, the choices underlying each model should be guided by their relevance to the specific research question. Despite the growing sophistication of MSK models, few sensitivity analyses have systematically examined how specific modeling choices may influence outcomes. Furthermore, the implications of added model detail on predictions and overall validity remain largely unexplored. A more detailed representation does not necessarily translate to improved accuracy, as the model may not be sensitive to certain refinements. In some cases, increased complexity may introduce new uncertainties or computational challenges without significantly enhancing predictions. This section explores different aspects of model complexity, reviewing current methodologies and highlighting key considerations for selecting an appropriate level of detail.

#### Specific Modeling Choices for MB, FE, and C Models

Great variability was found, first of all, regarding the specific modeling choices related to each approach.

MB models generally implement 3 rotational DoFs by default, eventually adding rotational stiffnesses (Fig. 1c-I) to mimic the passive behavior of intervertebral tissues [25, 40–43, 45, 51, 53, 54]. While translational DoFs are often neglected to simplify models—since axial and shear translations are small relative to rotations—the center of rotation in 3 DoF joints is crucial, as its position affects muscle and joint forces [126]. Ignoring translations can introduce errors in spinal kinematics and muscle forces [62] and in vivo studies show that coupled translational and rotational movements shift the center of rotation to reduce joint loads [127]. Although incorporating 6 DoFs could improve model accuracy, this is often not performed due to uncertainty and a lack of reliable data.

FE spine models are historically well accepted, as they allow a detailed representation of every spinal tissue with its unique mechanical behavior [128, 129]. Current FE MSK models represent the nucleus as an incompressible fluid-filled cavity and the anulus as an anisotropic material reinforced with fibers [27]. Although many simplifications in representing the soft tissues as 1D elements still remain, probably due to the high computational cost, nowadays technologies may further enhance realism by adopting a more involved representation of osteo-ligamentous tissues, describing them as fully deformable, therefore, ensuring more accurate assessments of biomechanically relevant variables [130]. Modern FE frameworks may also open the door to multi-scale modeling, potentially integrating tissue- and cell-level factors such as oxygen diffusion and cell density, thus advancing toward truly comprehensive MSK models.

Finally, given the inherent limitations of MB and FE modeling approaches, C models have become a topic of great interest in the MSK framework. Despite this, nowadays only a few original models are found in the literature [26, 66–70, 73, 74]. The most critical issue in CS is to ensure that the underlying MB and FE models are kinematically consistent, otherwise the coupling may not achieve the desired level of reliability [22]. This difficulty arises from the fact that MB and FE models are built on fundamentally different physical assumptions: MB models simulate the motion of rigid bodies, while FE models represent the deformation of flexible, continuum structures. As a consequence, the center of rotations, as well as the joint reaction forces and moments derived from MB models, do not directly correspond to those predicted by FE models that account for continuous stress and strain fields. This mismatch highlights a core limitation: since the models capture different mechanical behaviors, their outputs cannot be perfectly aligned, which complicates efforts to combine or compare them in an integrated simulation framework. Dynamic task poses even more challenges as inertia and acceleration are to be matched using different modeling strategies. To align the predictions of the two subsystem models in CSs, iterative adjustments were implemented in three models. However, no complete bidirectional coupling could be found in the literature regarding the thoracolumbar spine. In the case of HSs, a dedicated simulation platform with a unified solver enables the fully dynamic coupling of all MB and FE components, enabling the prediction of realistic loading conditions resulting from BW and muscle forces on the soft tissues of the spine to be investigated. However, the limited number of studies in this area suggests that this approach remains challenging, with some major compatibility difficulties yet to be solved.

#### Passive Elements are Required to Describe Spinal Load-Sharing

The spine’s passive behavior is governed by soft tissues (i.e., IVDs, ligaments, and FJs), which coordinate to share loads during specific movement. Ligaments alone can support over 30% of the force and up to 80% of the bending moment in flexion. FJs also play a key role in kinematics and load support [115, 131, 132]. Detailed FE models demonstrated the relevant contributions of these structures to load-sharing, showing that ligaments and facet joints bear substantial portions of internal forces and moments particularly during flexion and extension and that this distribution is further influenced by individual sagittal curvature [115, 133]. However, many MSK models focused only on the anterior column [25, 32–41, 44, 45, 47–50, 57–60, 62, 64], overlooking critical load-sharing from other structures, which limits their relevance in simulating conditions like LBP [115, 134]. When implemented, ligaments are often modeled as 1D elements [27, 42, 43, 46, 51–54, 61, 63]. For instance, Meszaros-Beller et al. used straight-line passive elements calibrated from literature data to capture their contribution during flexion [53, 135]. While 3D models are more computationally intensive, they may offer deeper understanding of soft tissue behavior and spine health [136].

FJs are often neglected or incompletely modeled in MB simulations, especially when considering flexion tasks [32, 33, 35, 36, 38, 42, 48, 52], despite their contribution to load-sharing may be relevant for other tasks [39, 44, 47, 49, 50]. While recent FE models included contact interactions [27, 63], detailed modeling remains limited, leading to poor reproducibility [137]. Although the integration of FJs is always feasible for all modeling approaches, in vivo evidence to support their load-bearing under physiological loads is lacking.

#### The Unloading Effect of IAP

The action of IAP has a relevant unloading impact on the lumbar spine, which has been demonstrated experimentally [138]. However, only 13 (32%) models (8 MB, 1 FE, 4 C) were found to account for this contribution. Among these, only 1 MB and 2 C models proposed a detailed prediction modeling scheme [51, 67, 69]. Liu et al. confirmed that higher spinal loads are found when IAP is not included in the models during flexion tasks and highlighted that this assumption also had an impact on global muscle forces, while the local ones remained unchanged [67]. Similarly, Guo et al. confirmed this unloading trend [51]. However, their approaches relied on a complex definition of IAP, whereas a simplified application of a force seems sufficient to model the IAP unloading effect [25, 37, 38, 40, 41].

#### Muscle Groups: Established Approaches for the Lumbar Spine and Emerging Strategies for Thoracic Regions

For the lumbar spine, consistent modeling choices for muscle architecture were supported by detailed anatomical studies [139–145]. In contrast, thoracic spine models varied widely, likely due to limited anatomical data for that region [146–148].

Among the existing models, there is a large variability in the number of fascicles the musculature is discretized into. In particular, MB models typically include a higher number of fascicles than FE models. For example, the median number of fascicles represented for multifidus is 25 for MB models, 6.5 for FE models, and 25 for C models (Fig. 4). Moreover, discrepancies were also observed among the same modeling approaches, with some models merging the various fascicles attached to the same levels into a single one [43, 48, 58, 68]. While this simplification is often motivated by functional rationales—such as ensuring fascicles span origin and insertion points covered by the muscle—it remains unclear what the appropriate level of discretization or how many fascicles a muscle should be divided into. Although simplifying the model can be justified by the need for computational efficiency, precise justifications for the number of fascicles chosen for specific muscle groups are often missing. This is particularly important, as the number of fascicles used in a model has been shown to influence recruitment patterns and, therefore, could significantly impact the interpretation of simulation results [149]. In general, muscle groups to be implemented at the lumbar levels are well-established, while further studies should be performed for the thoracic area.

#### Muscle Insertion Sites: Assumptions and Sensitivity Analysis on Their Positioning

Muscles in spine models are represented by selecting the positions of the origin and insertion sites of each fascicle. Precise coordinates of these sites were provided by only 9 articles [25, 32, 33, 36, 40, 46, 48, 58, 69], but anatomical data weren’t always available.

However, the significance of these positions must be considered. For instance, Bayoglu et al. conducted a sensitivity analysis on muscle origin and insertion sites to assess their impact on biomechanical variables. They found that small changes in the attachment sites of certain muscles (i.e., the quadratus lumborum and longissimus thoracis pars lumborum) lead to significant differences in predicted muscle and shear forces [77]. Given the importance of muscle fascicle architecture, it is recommended that future studies share a comprehensive dataset of their coordinates and conduct sensitivity analyses to assess the effects of varying muscle attachment points on model’s predictions.

#### Muscle Models: Detailed at Tissue Level, Simplified at Organ Level

After selecting muscle positions, their behavior must be defined. Many models use simple force actuators with physiological constraints [25, 26, 32–35, 37, 38, 40, 43, 46–48, 52, 55, 57, 60, 61, 66–68], while others use Hill-type model for a more detailed depiction of the musculotendon system [150]. Only one model described muscles as 3D structures generating contraction and represented them as fluid-filled cavities, where intramuscular pressure could be extracted and correlated to muscle forces [65]. Hill-type model requires multiple muscle parameters, such as cross-sectional area, stiffness, sarcomere lengths, pennation angle, and specific tension, and many of these lack sufficient data and thus are often assumed [151] or adapted from other models. While cross-sectional area clearly affects loading and activation [152–154], the impact of other parameters is still debated. Malakoutian et al. found that all the parameters apart from the pennation angle significantly influence L4-L5 IDP and interact with each other [151]. Accurate force-length modeling thus depends on these variables that may also be subject-specific, but due to their variability, more in vivo studies are needed. A stiffness-based method offers a simpler alternative by reducing reliance on detailed muscle parameters.

#### From Kinematics to Solution Approach

Whether kinematics is used as input or verified as output, assumptions about motion distribution—this concept is often recalled as ‘lumbar rhythm’—across functional spinal units should be clearly stated. This rhythm influences how movement is distributed across spinal segments, especially during deep flexion, significantly impacting internal forces like compression and shear [117]. The literature review highlighted that many models simplify [25, 40, 41, 71, 72] or assume fixed distributions of intervertebral motion without detailed justification [39, 46, 49, 50, 52, 59, 62, 64, 66–68].

To address motion and force estimation, FD, ID, or FD-assisted approaches can be used. In practice, these approaches may be ideally used interchangeably, with the choice largely deriving from the type and quality of available input data. It is important to notice that ID-based models solve an inherently indeterminate problem by applying optimization strategies to determine the most plausible solution. Early ID models relied on static optimization, assuming that each time point could be solved independently [155]. Among the optimization criteria, minimizing muscle stresses has emerged as the most common and physiologically grounded method [25, 27, 34, 37, 39–41, 43, 44, 46–50, 52, 59, 62, 64, 66–68, 71, 72], especially when constraints on maximum allowable loads are included [156]. In fact, optimization schemes aim to replicate the central nervous system’s decision-making during movement. In the case of minimizing muscle stresses, fatigue is considered by inserting the polynomial term (e.g., cubed muscle stresses) [155, 157] and more intuitively speaking, employing larger muscles to produce forces is preferred and results in physiological patterns [158]. One work proposed two novel criteria based on minimizing and maximizing intramuscular pressure and highlighted its role in muscle recruitment and potential load-sharing with surrounding tissues [76].

Despite being fundamental for spine biomechanics, spinal stability was directly addressed in only 6 original MSK studies [48, 57–60, 69]. Analyzing spinal stability provides valuable insights into the role of muscle function and helps identify sources of instability, which are closely associated with spinal injuries and pathologies [10]. In general, posture was found to impact the spinal system and its stability margins [57, 59, 69, 159], and abdominal co-activation was also found to play a role [48, 114]. Overall, current findings emphasize the importance of understanding spinal stability as a dynamic balance between muscle activation and tissue loading. Further research is needed to understand how posture and load distribution affect the interaction between the active (muscular) and passive (structural) components of the spine [114, 159].

### Choosing Personalization Level

Models of the human MSK system can be personalized to better reflect individual anatomy and biomechanics, offering more accurate and clinically relevant insights. However, many current approaches rely on ‘*average’* models, which often overlook the natural interpersonal variability in features such as spine curvature and muscle attachment sites, potentially reducing their realism and usefulness. Truly *subject-specific* models demand extensive information, often unavailable. Such data is crucial not only for understanding variability but also for identifying which model features are most sensitive and for establishing meaningful reference values and ranges.

#### Reference Population: Generic, Mean, and Subject-Specific Model

A clear definition of the targeted reference population is a fundamental step in the modeling process and should be established at the outset. More broadly, selecting the appropriate level of personalization is a key consideration in model development. Three general levels of individualization have been proposed, depending on how the predictive accuracy of the model is quantified against the available evidence [28]. While generic models (level 1) should be compared only to the range of observed values, mean models (level 2) are relevant to describe a standard generalized individual from a wider (generally physiological) population, and subject-specific models (level 3) represent the characteristic traits of one specific subject (typically pathological) [28]. While levels 1 and 2 are useful for general trends, level 3 is essential for assessing patients with alignment deformities.

The generic models analyzed for this review were found to fit within the same range of mean models representing a standard healthy young man [34, 35, 37, 38, 61]. On the other hand, subject-specific models covered a wider range of cases [46, 49, 50, 52, 53, 64, 69–72]. However, only four models representing females were found [44, 52, 58, 59, 160], thus highlighting a strong bias in biomechanical evaluations, with males representing the default target for research [160]. In general, morphological comparisons could be carried out just in terms of general aspects (height, weight, age), as just a few works reported an exhaustive description of the sagittal alignment (e.g., lumbar lordosis, pelvic incidence, sacral slope) of patients [39, 49, 52, 161]. However, studies on subject-specific models highlighted the significant influence of individual spinal curvature on biomechanical values like vertebral loading, shear, and muscle forces [161–164]. To improve accuracy, future models should precisely describe sagittal alignment parameters.

#### Body Weight Distribution Should be Tailored for Obese Patients

To achieve the desired kinematics for a given task, the spine must balance gravitational loads with appropriate muscular forces to maintain stability and control. Therefore, accurately implementing BW in MSK spine models is crucial for correctly estimating both muscle forces and the resulting spinal loads [161]. Standard BW distributions, like those from Pearsall et al. [75], work for average individuals, but adjustments are needed for specific populations, such as patients with obesity. Several studies have shown that in obese individuals, scaling methods must be applied with particular care, as inaccuracies in segmental mass distribution can significantly affect spinal loading during functional tasks and alter joint and ground reaction force predictions during gait [165, 166].

Ghezelbash et al. accounted for adipose tissue in obese patients, revealing the notable center of mass shifts at lumbar levels [167], while Liu et al. used subject-specific data to highlight differences at the thoracic level [168]. Recent advances in machine learning have enabled the development of efficient, anthropometry-based neural network models that offer a promising and practical tool for predicting subject-specific trunk mass and center of mass in musculoskeletal modeling [169].

### Challenges and Future Directions

The MSK modeling framework presents a high degree of complexity, and the literature reflects this through a wide range of diverse approaches aimed at understanding, modeling, or facing it. Despite the variety of available solutions, the present review revealed several recurring challenges across studies. These common issues, along with emerging trends, point toward promising future directions, briefly summarized in the following list.

- **Sensitivity analyses**: Even though a variety of modeling choices are nowadays available for MSK modeling (e.g., joint properties, number of muscle fascicles), sensitivity analyses on the effects of such modeling assumptions are scarce in the literature [77]. Such analyses are essential to evaluate the influence of varying modeling choices and align with the principles outlined in the ASME V&V guidelines [23]. Realistically, it is unfeasible to test all possible modeling configurations due to computational limitations and the current lack of extensive reference datasets. To address this, comprehensive datasets reflecting interpersonal variability and advanced computational methods—such as machine learning and stochastic simulations—are needed to assess the impact of model assumptions and improve personalization and predictive accuracy. Probabilistic simulations combined with statistical shape modeling may be used to evaluate population-wide variability, as has been proposed for rib material properties and anatomical shapes [170].
- **Validation**: Validation of MSK models remains a major challenge due to the limited availability of comprehensive in vivo data and the lack of standardized procedures. Every model must be validated against reference data to ensure its reliability for further analysis, yet the lack of standardized validation frameworks makes consistent and meaningful comparisons across studies challenging. The adoption of structured guidelines [23] could help address this issue by offering a unified approach also in the spine models, enhancing transparency, reproducibility, and comparability [171].

The literature review highlighted that in vivo and in silico data were mostly used and some common references have been identified, particularly predicted IDPs are mostly compared to those reported by in vivo studies [9, 83]. The validation of muscle forces in single fascicles can’t be performed with comparison to experimental measurements; however, comparisons can be made using EMG data, for example, in terms of percentage of contraction [102, 105]. These signals are acquired via superficial electrodes and provide general information about muscle activation, but lack the precision needed for detailed or fine-scale validation. As a consequence, some works proposed in silico comparators as a reference. Although the robustness of in silico comparators is still debated [30], any effort to compare results to other available works is appreciable. For instance, Bruno et al. extensively validated both muscle activation patterns and IDPs, showing high correlations to in vivo measurements [39] and setting a benchmark for future studies to reference. In this context, emerging non-invasive methods, like MRI-based measurements and digital volume correlation [172], offer promising tools for building diverse datasets and supporting AI-driven validation strategies. Finally, clear documentation of modeling choices and validation processes is essential to improve transparency.

- **Simulated tasks**: Most MSK models of the spine focus on relatively mild, simplified tasks such as upright standing or controlled flexion [25–27, 34, 36, 39–44, 46, 47, 49–56, 59, 61, 62, 64, 66–69, 71, 72]—typically up to 30 degrees—often without significant external loading. While these scenarios are useful for baseline analyses, they do not reflect more demanding tasks, where spinal complications are more likely to arise. Pathological conditions and postoperative issues typically occur under complex, combined loading scenarios, such as lifting with trunk rotation, stair climbing, or dynamic activities like running or sports. In the next years, there is a strong need to expand spinal biomechanical analyses to include these more realistic and challenging daily tasks, which will better represent worst-case conditions and improve the clinical relevance of model predictions. For instance, the study of MSK injuries among workers responsible for material handling and lifting tasks has already gained increasing interest, and the contribution of MSK modeling could be fundamental for injury prevention. It has been demonstrated that MB tools allow for accurate assessment of spinal loading and ground reaction forces in real occupational environments where force plates are not available [173, 174] and can be used to propose effective engineering interventions to prevent injuries [175].
- **Personalization of joint properties**: Joint properties, such as rotational stiffness for specific tasks, are often assigned using generic values [36, 52, 55], but personalization of these parameters is crucial for realistic MSK simulations [176]. A mismatch between joint stiffness and observed kinematics—such as simulating a highly flexible spine using overly stiff joint properties—can lead to unrealistic muscle forces, as the model compensates by overactivating muscles to achieve the same ROM. As the interplay between joint stiffness and range of motion was demonstrated [177], their interaction should be carefully evaluated. Despite efforts to personalize spinal kinematics, joint properties are rarely individualized due to a lack of non-invasive experimental methods. While some approaches calibrate curve flexibility using radiographic data in clinical cases like adolescent idiopathic scoliosis or adult spinal deformity, a broader integration of sensitivity analyses and uncertainty quantification, as encouraged by the ASME V&V framework [23] is needed.
- **Whole torso biomechanical calibration**: Current MSK models treat the spine as a standalone structure, represented as a stack of isolated functional spinal units, overlooking the continuous nature of the trunk. However, surrounding soft tissues (e.g., abdominal organs, skin, and adipose tissue) may contribute to resisting motion and play a role in distributing forces across spinal segments, especially during deep flexion or complex movements. Some preliminary numerical models that attempted to model the continuity of the trunk were found in the literature [65, 178]. To improve model fidelity, stepwise calibration should consider the entire torso rather than just individual functional spinal units, potentially supported by new in vivo measurement techniques.
- **Personalized properties**: Despite the growing interest in high levels of personalization for large cohorts, most cohort studies still rely on only partially individualized modeling approaches, like tuning for motion, muscle strength, or spinal curvature, without full subject specificity. In contrast, highly detailed models like Bayoglu’s are based on a single subject, offering depth, but lacking broader applicability [46, 145, 147]. Moreover, current efforts to establish models of pathology often begin with a physiological baseline, which is an imprecise approach, as pathological models require distinct assumptions. To build more representative models—particularly for properties like muscle force or joint behavior—we urgently need more targeted, population-specific measurements. Furthermore, longitudinal studies could play a critical role by tracking individual biomechanical and physiological changes over time, enabling the development of models that better reflect both the onset and progression of MSK conditions.
- **The need for Open Science**: Open Science is essential to improve spine models, especially by enhancing transparency in model construction, development, and verification. Yet, in most models, significant modeling decisions are not well-documented, limiting reproducibility and comparability. To remedy that, there is an increasing need for open-access repositories in which models can be freely shared, reused, and adequately annotated: SimTK [179], DemoA [180], and ArtiSynth [181] are virtuous examples. Projects like these foster scientific rigor, facilitate reproducibility, and stimulate innovation by allowing everyone to leverage clearly documented, tested resources.

In conclusion, MSK spine modeling presents a high degree of complexity and requires careful consideration of numerous biomechanical factors. To ensure that these models are trusted and effectively applied within the spine research framework, it is essential to employ modeling strategies that are validated, transparent, and well documented. Thanks to rigorous approaches, researchers can confidently rely on these models to address specific scientific questions and bring further developments in this area.

## Supplementary Information

Below is the link to the electronic supplementary material.

Supplementary file1 (PDF 299 KB)

## Data Availability

The authors confirm that the data supporting the findings of this study are available within the article [and/or] its supplementary materials.
