# Supplementary material for: Advances in Musculoskeletal Modeling of the Thoraco-Lumbar Spine: A Comprehensive Systematic Review
Source: Ann Biomed Eng. 2025 Sep 5;53(11):2883–910. doi: 10.1007/s10439-025-03818-8 (PMC12575568; doi:10.1007/s10439-025-03818-8)
Supplement: Supplementary file 1 — Supplementary file1 (PDF 299 KB) [file 10439_2025_3818_MOESM1_ESM.pdf]

Advances in musculoskeletal modeling of the thoraco-lumbar spine: a comprehensive systematic review

Linda Carpenedo[1]*, Dominika Ignasiak[2], Robin Remus[3], Luigi La Barbera[1]

[1] LaBS - Department of Chemistry, Materials and Chemical Engineering "Giulio Natta", Politecnico di Milano, Piazza Leonardo da Vinci 32, 20133 Milano, Italy

[2] Institute for Biomechanics, ETH Zurich, Zurich, Switzerland

[3] Chair of Product Development, Department of Mechanical Engineering, Ruhr-University Bochum, Bochum, Germany

*Corresponding author

E-mail address: linda.carpenedo@polimi.it

Table 1: Reasons for inclusion of non-original works

Ref.	Original model	Reason for inclusion
2004, El-Rich	1998, Kiefer	Considerations on spinal stability
2005, El-Rich	1998, Kiefer	Considerations on spinal stability
2010, Bresnhan	2007, De Zee	Impact of resection (different physiological cross-sectional area) of paraspinal muscles deriving on muscle activity
2016, Arshad	2007, De Zee	Impact of lumbar spine rhythm and intra-abdominal pressure on predicted loads and muscles forces (in particular during flexion)
2016, Malakoutian	2018, Malakoutian	Effect of simulated muscle damage (different physiological cross-sectional area) on post-operative spinal loading at the adjacent levels to a spinal fusion
2016, Ghezelbash	2016, Ghezelbash	Effects of sex, age, body height, and weight on spinal loads
2016, Zander	2007, De Zee	Impact of the position of centers of rotations on the activities of lumbar muscles and the joint forces between vertebrae
2017, Bruno	2015, Bruno	Subject-specific musculoskeletal models of the thoracolumbar spine
2018, Ignasiak	2016a, Ignasiak	Impact of sarcopenia (different physiological cross-sectional area) on spinal loading and on muscle activity
2019, Bassani	2016a, Ignasiak + 2007, De Zee	Effects of sagittal alignment parameters on intervertebral loads in the lumbar spine and muscle activation
2019, Bayoglu	2019, Bayoglu	Sensitivity analysis on muscles' attachment sites
2022, El Bojairami	2020, El Bojairami	Further development of the model (implementation of different optimization criteria)
2022, Malakoutian	2018, Malakoutian	Relevance and effects of the muscle force-length curve and the correlated parameters
2023, Bahramian	2016a, Ignasiak + 2007, De Zee	Effects of subject-speicific modeling of obesity for musculoskeltal predictions
2023, Liu	2018, Liu	Mass-scaling patient-specific technique
2023, Meszaros-Beller	2023, Meszaros-Beller	Impact of passive spinal structures on joint loads
2023, Manohoran	2012, Christophy	Application of a musculoskeletal model to study low back pain
2024, Caimi	2007, De Zee	Impact of different optimization criteria on muscle force prediction
2024, Daroudi	2016a, Ignasiak + 2007, De Zee	Applicability of MSK modeling to study complex tasks: load reaching and handling activities
2024, Dehghan	2016a, Ignasiak + 2007, De Zee	Applicability of MSK modeling to study complex tasks: load handling activities
2024, Khosrozadeh	2016a, Ignasiak + 2007, De Zee	Applicability of MSK modeling to study complex tasks: lifting tasks
2024, Lariviere	2016, Ghezelbash	Impact of changes in the spinal profile on biomechanical
2024, Lerchl	2023, Lerchl	Effects of morphological individualization on spine biomechanics
2025, Hulleck	2016a, Ignasiak + 2007, De Zee	Effects of body mass distribution during gait for normal and obese patients
2025, Remus	2023, Remus	Considerations on spinal stability: Implementation of torso soft tissue for interaction simulations with orthoses

Table 2: Detailed muscle fascicles for MB models. MF=Multifidus (L=Lumbar, T=Thoracic), ES=Erector Spinae, IP=Iliopsoas, RA=Rectus Abdominis, EO=External Oblique, IO=Internal Oblique, QL=Quadratus Lumborum, LD=Latissimus Dorsi, ISP=Interspinales, ITS=Intertransversarii, TSP=Transversospinalis, SP=Spinalis, ST=Serratus

**Reference**	**Number of fascicles per side**																		**Number of fascicles (both sides)**
	**MF**	**ES**				**IP**	**RA**	**EO**	**IO**	**QL**	**LD**	**ISP**	**ITS**	**TSP**		**SP**	**ST**	**Fascicles for less frequent muscle groups**	
		**IC**		**LT**										**RT**	**SMP**				
		**PL**	**PT**	**PL**	**PT**														
1995, Stokes	20L, 5T	8	8	1	12	11	1												132
2007, De Zee	19	29				11	1	6	6	5									154
2010, Iyer		12				5	8	8	4	4	10			12			6	4 Pectoralis Major, 6 Trapezius	158
2011, Han	20	12		24		6	4	3	3	5	7	6	10	11		3	2		232
2012, Christophy	25	12		26		11	1	6	6	18	14								238
2012, Han	62	24		34		22	1	6	6	10	10	12	22	22	18		4	5 Transversus Abdominis	516
2012, Park	20L, 12T	4	8	5	12	11	1	6	6	5									180
2015, Bruno	37	12		26		11	1	8	6	18	14						10	38 Internal Intercostales, 38 External Intercostales, 5 Transversus Abdominus; 20 Scapular, 14 Trapezius, 8 Shoulder Muscles, 3 Neck Muscles, 7 Cervical Multifidus	248
2015, Huynh	19	4	8	5	12	11	1	6	6	5									154
2015, Khurelbaatar	5L, 4T	15				6	1	5	6	3		6	13	35	10			22 Cervical Muscles	218
2015, Meng	25	12		26		11	1	6	6	18	14								238
2015, Rupp	25	4		5		11	1	6	6				4						124
2015, Senteler	25	12		26		11	1	6	6	18	14								238
2016, Dao	25	12		5		11		2	3	18									152
2016a, Ignasiak	19L 24T	37				11	1	6	6	5					27	3		46 Muscles Stabilizing Ribs and Sternum, 29 Levator Costarum (Brevis and Longus) and Subcostalis, 13 Transversus Thoracis	454
2016b, Ignasiak	19L, 24T	37				11	1	6	6	5					27	3		46 Muscles Stabilizing Ribs and Sternum, 29 Levator Costarum (Brevis and Longus) and Subcostalis, 13 Transversus Thoracis, 27 Intercostales	508
2017, Bassani	De Zee, 2007																		
2018, Actis	Christophy, 2012																		
2018, Malakoutian	25	4	8	5	21	11	1	6	6	18									210
2019, Bayoglu	15L, 49T	6	3	23		10	3	7	6	8	5			26	4	6	3	10 Transversus Abdominis, 6 Intercostales Externis, 5 Intercostales Interni, 13 Levatores Costarum, 2 Rhomboidus Major, 1 Rhomboideus Minor, 8 Serratus Anterior, 3 Serratus Posterior Superior, 1 Subclavious, 8 Subcostales, 6 Transversus Thoracis, 9 Trapezius, 75 Cervical Fascicles, 30 Intercostales and Levatores Costarum at the	552

																		Ribcage Levels	
2019, Higuchi	17	8	6	32		10	3	8	12	5		7	12	34	6	4			328
2019, Kamal	5L, 5T	6	1	6	1	5	1	6	6	4									92
2021, Guo	25	38				11	1	8	6	18									214
2021, Fasser	25	12	1	26	1	5	1	6	6	18	14								230
2021a, Favier	25	12		26		11	1	6	6	18	14								238
2022, Lerchl [	25	12	1	26	1	11	1	1	1	19		5							206
2023, M.-Beller	25L, 24T	4	8	5	21	11	4	6	6			16	4		5	8			294
2023, Mo	25	12		26		11	1	6	6	18	14								238

Table 3: Detailed muscle fascicles for FE models. MF=Multifidus (L=Lumbar, T=Thoracic), ES=Erector Spinae, IP=Iliopsoas, RA=Rectus Abdominis, EO=External Oblique, IO=Internal Oblique, QL=Quadratus Lumborum, LD=Latissimus Dorsi, ISP=Interspinales, ITS=Intertransversarii, TSP=Transversospinalis, SP=Spinalis, ST=Serratus

**Reference**	**Number of fascicles per side**																		**Number of fascicles (both sides)**
	**MF**	**ES**				**IP**	**RA**	**EO**	**IO**	**QL**	**LD**	**ISP**	**ITS**	**TSP**		**SP**	**ST**	**Fascicles for less frequent muscle groups**	
		**IC**		**LT**										**RT**	**SMP**				
		**PL**	**PT**	**PL**	**PT**														
1997, Kiefer	1 local, 1 global																		4
1998, Kiefer	5	5	1	5	1	5	1	1	1	4						1			60
2006, Arjmand	5	4	1	5	1	5	1	1	1	4									56
2008, Kim K.	20L, 12T	4	8	5	12	11	1	6	6	5		6	10	11					234
2014, Kim B.S.	20	12		24		6	4	3	3	5	7	6	10	11		3	2		232
2015, Ghezelbash	5	4	1	5	1	5	1	1	1	4									56
2015, Tourmaidou	8	2		3	4	6													46
2016, Ghezelbash	20	4	3	5	3	5	1	4	6	8						4			126
2020, El Bojairami	3D			3D		3D					3D		3D						3D
2021, Rajaee	5	4	1	5	1	5	1	1	1	4									56

Table 4: Detailed muscle fascicles for C models. MF=Multifidus (L=Lumbar, T=Thoracic), ES=Erector Spinae, IP=Iliopsoas, RA=Rectus Abdominis, EO=External Oblique, IO=Internal Oblique, QL=Quadratus Lumborum, LD=Latissimus Dorsi, ISP=Interspinales, ITS=Intertransversarii, TSP=Transversospinalis, SP=Spinalis, ST=Serratus

**Reference**	**Number of fascicles per side**																		**Number of fascicles (both sides)**
	**MF**	**ES**				**IP**	**RA**	**EO**	**IO**	**QL**	**LD**	**ISP**	**ITS**	**TSP**		**SP**	**ST**	**Fascicles for less frequent muscle groups**	
		**IC**		**LT**										**RT**	**SMP**				
		**PL**	**PT**	**PL**	**PT**														
2018, Liu	19	4	8	5	12	11	1	6	6	5								17 Iliacus	188
2018, Khoddam-Khorasani	5	4	1	5	1	5	1	1	1	4									56
2019, Liu	19	4	8	5	12	11	1	6	6	5									154
2021b, Favier	2021a, Favier																		238
2021, Kumaran	2015, Bruno																		248
2021, Panico	19L, 24T	37				11	1	6	6	5					27	3		46 Muscles Stabilizing Ribs and Sternum, 29 Levator Costarum (Brevis and Longus) and Subcostalis, 13 Transversus Thoracis, 54 Intercostales	508
2023, Remus	25	4	8	5	21	11	1	6	6	18	14							10 Transversus Abdominis	258
2023, Xu	25	12		26						18									162
